# Endobronchial ultrasound-guided transbronchial needle aspiration facilitating diagnosis of sarcoidosis in a breast cancer patient with multiple lymphadenopathy: a case report

**DOI:** 10.1186/s13256-022-03428-1

**Published:** 2022-05-19

**Authors:** Yuka Oride, Yumiko Koi, Tatsunari Sasada, Keiko Kajitani, Masahiro Ohara, Tomohiro Kondo, Yutaka Daimaru, Shingo Kawamura

**Affiliations:** 1 Department of Breast Surgery, JA Hiroshima General Hospital, 1-3-3 Jigozen, Hatsukaichi, Hiroshima 738-8503 Japan; 2 Department of Breast Oncology, National Hospital Organization Kyushu Cancer Center, 3-1-1 Notame, Minami-ku, Fukuoka, 811-1395 Japan; 3 Department of Respiratory Medicine, JA Hiroshima General Hospital, 1-3-3 Jigozen, Hatsukaichi, Hiroshima 738-8503 Japan; 4 Section of Pathological Research and Laboratory, JA Hiroshima General Hospital, 1-3-3 Jigozen, Hatsukaichi, Hiroshima 738-8503 Japan; 5 Suzugamine Imanaka Clinic, 4-2-31, Inokuchi, Nishi-ku, Hatsukaichi, Hiroshima, Hiroshima 733-0842 Japan

**Keywords:** Breast cancer, Sarcoidosis, Endobronchial ultrasound-guided transbronchial needle aspiration

## Abstract

**Background:**

Sarcoidosis is a benign systemic granulomatous disorder of unknown etiology. Cell-mediated immunity disorder is often found in sarcoidosis patients, and an association between malignant tumors and sarcoidosis has been suggested. Sarcoidosis and malignant disease can occur simultaneously or sequentially, leading to misdiagnosis and mistreatment. Sarcoidosis is diagnosed clinically, radiologically, and histologically. We report herein a case of sarcoidosis diagnosed by endobronchial ultrasound-guided transbronchial needle aspiration from the mediastinal lymph nodes of a breast cancer patient.

**Case presentation:**

The patient was a 70-year-old Asian woman who presented with right breast tumor. A 20-mm movable mass was identified in the inferolateral quadrant of the right breast, and mammography revealed a spiculated mass with calcification. Ultrasonography revealed a mass with internal hypoechogenicity, and biopsy revealed estrogen receptor-positive, human epidermal growth factor receptor 2-positive invasive ductal carcinoma. Positron emission tomography/computed tomography showed multiple lymphadenopathy including mediastinal lymph nodes, with fluorodeoxyglucose accumulation in those nodes suggesting breast cancer metastases. Endobronchial ultrasound-guided transbronchial needle aspiration of a mediastinal lymph node revealed noncaseous epithelioid granuloma. Due to a history of uveitis and elevated soluble interleukin 2 receptor, lymphadenopathy due to sarcoidosis and stage IIA breast cancer were diagnosed. Right partial mastectomy and axillary lymph node dissection were performed after preoperative chemotherapy. No exacerbation of sarcoidosis symptoms has been observed during treatment.

**Conclusion:**

We report a case of breast cancer in which sarcoidosis could be diagnosed based on endobronchial ultrasound-guided transbronchial needle aspiration, a history of uveitis, and elevated soluble interleukin 2 receptor despite fluorodeoxyglucose positron emission tomography/computed tomography suggesting multiple lymph node metastases. This report emphasizes the importance of differential diagnosis of lymph node involvements in cancer patients.

## Background

Sarcoidosis is a benign systemic granulomatous disorder of unknown etiology, characterized by the formation of noncaseating epithelioid cell granulomas in the lungs, mediastinum, and lymphatic system but also potentially in the salivary glands, heart, nervous system, joints, and various other organs [1]. Disorders of cell-mediated immunity are often found in sarcoidosis patients, and Brincker and Wilbek [2] reported in 1974 that malignant tumors are frequently associated with sarcoidosis.

In follow‑up computed tomography (CT) or positron emission tomography/computed tomography (PET/CT) for detection of primary tumors or metastases, sarcoidosis can mimic breast cancer recurrence or metastatic progress, leading to misdiagnosis and incorrect treatment [3–6]. Although a diagnosis of sarcoidosis is established on the basis of compatible clinical and radiological findings, histological evidence for noncaseating epithelioid cell granuloma is often required to differentiate sarcoidosis from metastatic disease [7]. Breast cancer continues to be the most frequently diagnosed female cancer worldwide (2.26 million cases) [8]. Overall survival has improved in recent decades with new therapy options based on accurate diagnosis [9].

We report herein a case of sarcoidosis diagnosed using endobronchial ultrasound-guided transbronchial needle aspiration (EBUS-TBNA) from a mediastinal lymph node in a patient with breast cancer before treatment of the cancer.

## Case presentation

**Fig. 1:**
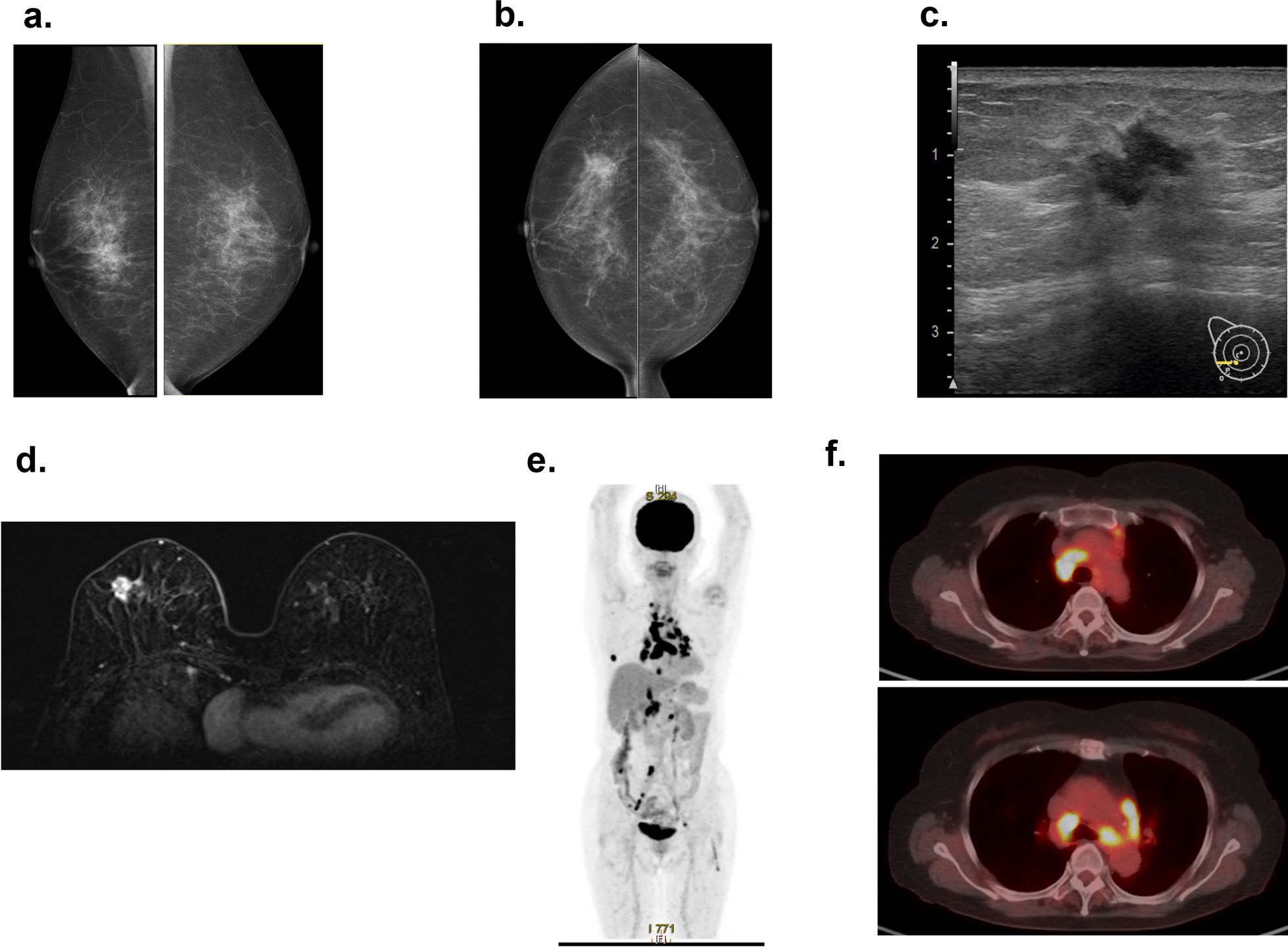
Preoperative imaging findings. **a** Mediolateral oblique-view mammogram. **b** Craniocaudal-view mammogram. A spiculated mass (A) with segmental and linear calcification is recognized in the right breast. **c** Ultrasonogram. A rough-bordered mass with internal hypoechogenicity is apparent in the inferolateral quadrant of the right breast. **d** MRI with early gadolinium enhancement. A smooth, round mass with rim enhancement is recognized in the inferolateral quadrant of the right breast. **e** Positron emission tomography. Accumulation of FDG is evident in the primary mass in the right breast, a right axillary lymph node, and multiple lymph nodes in the right supraclavicular fossa, mediastinum, bilateral hilar, dorsal pancreatic head, abdominal aorta, right common iliac region, and bilateral external iliac regions. **f** Positron emission tomography/computed tomography. Intense FDG uptake is shown in enlarged paratracheal lymph nodes

**Fig. 2:**
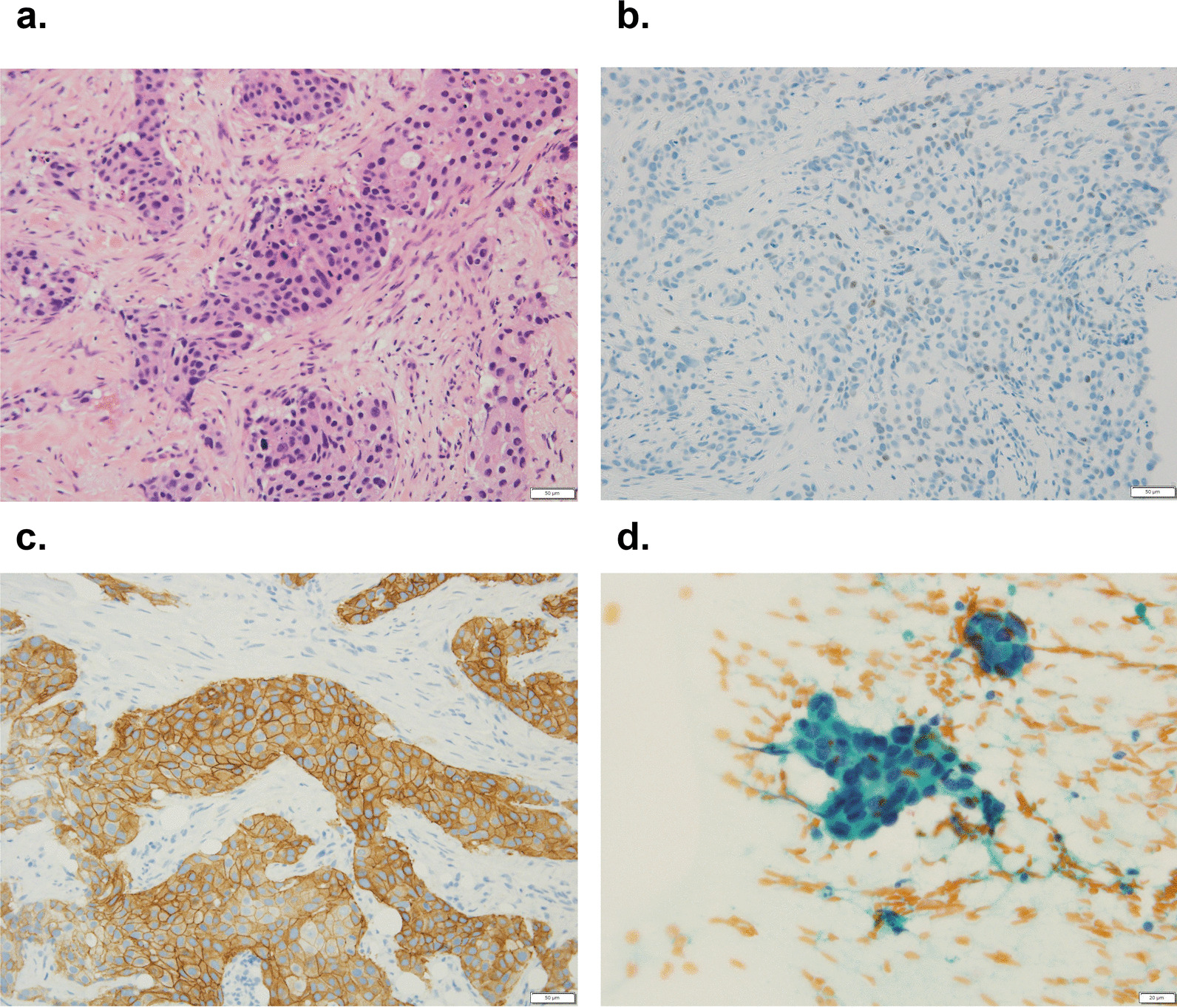
Preoperative histopathological findings. Histopathological findings of the right breast from core needle biopsy [**a** hematoxylin and eosin (HE) ×200]. Immunohistochemistry study for ER and HER2 (**b** ER ×200 and ×400, c: HER2 ×200). The cytology of the right axillary lymph node from fine needle aspiration cytology (**d** Papanicolaou ×400). Atypical ductal cells, ER-weakly positive (1–5%) and HER2-positive, formed solid nest with sheet-like growth. Atypical epithelial clusters with hyperchromatic nuclei were seen in red blood cells and lymphocytes

**Fig. 3:**
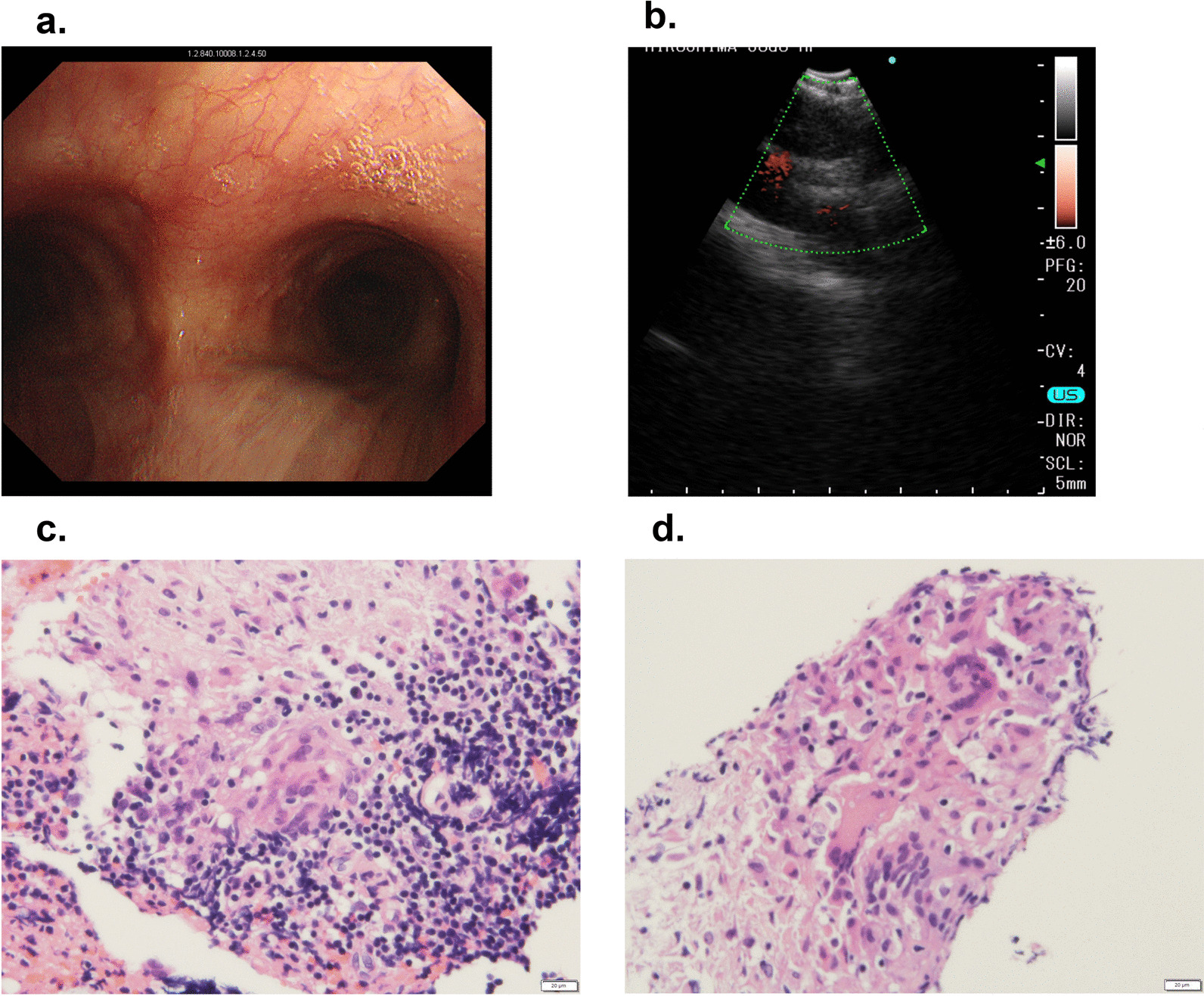
EBUS-TBNA findings. **a** Endoscopic view of the tracheal bifurcation. **b** EBUS ultrasound image of the lesion at the tracheal bifurcation. **c**, **d** Histopathological findings of biopsy samples directly obtained by EBUS-TBNA (HE ×400). A swollen lymph node is recognized at the extratracheal region on ultrasound images despite a normal appearance from the trachea. Lymph nodes have been replaced by noncaseous epithelioid granulomas and multinucleated giant cells

The patient was a 70-year-old Asian woman who had been referred to our hospital complaining of a right breast tumor. Physical examination revealed a 20-mm movable hard mass within the inferolateral quadrant of the right breast with no findings on the overlying skin. Mammography revealed a spiculated mass with segmental and linear calcifications in the mediolateral portion of the right breast (Fig. 1a, b). Ultrasonography revealed a rough-bordered mass showing internal hypoechogenicity and measuring 16 × 12 × 11 mm^3^ in size, in the inferolateral quadrant of the right breast (Fig. 1c). Magnetic resonance imaging showed a round mass, measuring 14 × 13 × 11 mm^3^ in size with rim enhancement (Fig. 1d). The tumor was diagnosed as estrogen receptor-positive, human epidermal growth factor receptor 2 (HER2)-positive invasive ductal carcinoma from core needle biopsy (Fig. 2a–c). Fine needle aspiration cytology of an axillary lymph node measuring 12 mm in diameter revealed the presence of malignant cells (Fig. 2d). PET/CT also showed fluorodeoxyglucose (FDG) accumulation in the right breast primary [maximum standardized uptake value (SUVmax) 11.5] and right axillary lymph node (SUVmax 2.1). Multiple lymph nodes in the right supraclavicular fossa, mediastinum, and around the bilateral hilar, dorsal pancreatic head, abdominal aorta, right common iliac region, and bilateral external iliac region appeared swollen and positive for FDG accumulation (SUVmax 17.0), suggesting distant lymph node metastases from breast cancer (Fig. 1e, f). Differential diagnoses for multiple lymphadenopathy were required because of the large dissociation between the primary breast cancer and locoregional and distant metastases. Lymph node involvement by malignant tumor, especially malignant lymphoma, and granulomatous disease could be enumerated as differential diagnoses. Suitable lymph nodes that appeared swollen and positive for FDG accumulation near the body surface could not be found for biopsy. For histological diagnosis of multiple lymphadenopathy, EBUS-TBNA of mediastinal lymph nodes was performed (Fig. 3a, b). Histological findings revealed noncaseous epithelioid granulomas and multinucleated giant cells in mediastinal lymph nodes (Fig. 3c, d). Tuberculosis had not been suspected strongly because CT scan did not reveal any exudative lesion in the lung field. Caseous granulomas were not found in all areas on the biopsy sample; therefore, acid-fast bacillus staining was omitted. This patient had been diagnosed with uveitis of unknown cause 5 years before. Systemic disease including sarcoidosis had been suspected, and necessary screening examining unknown details had been performed. The serum angiotensin-converting enzyme was 9.9 U/mL in therange of 7–25 U/mL, and soluble interleukin-2 receptor (sIL-2R) was increased to 627 U/mL (reference range 122–496 U/mL) just before this EBUS-TBNA. Based on a history of uveitis, the elevated concentration of serum soluble interleukin-2 receptor, and the histological findings, we diagnosed sarcoidosis lymphadenopathy and T1cN1M0 stage IIA breast cancer. Though further lymph nodes should be examined by EBUS-TBNA or mediastinoscopy to completely exclude malignant lymphoma, sarcoid-like reaction, and granulomatous disease, we reached these diagnoses comprehensively.

Right partial mastectomy and axillary lymph node dissection were performed after preoperative chemotherapy (four cycles of trastuzumab, pertuzumab, and docetaxel, followed by four cycles of epirubicin and cyclophosphamide). Histological examination of the surgical specimen revealed disappearance of the tumor. She had completed postoperative radiotherapy to the right breast and the right supraclavicular fossa and 14 cycles of adjuvant trastuzumab therapy. The endocrine therapy with letrozole has continued. She remained free of symptoms of sarcoidosis exacerbation at 2 years after her breast surgery, visiting cardiology, respiratory medicine, and ophthalmology every 3 months.

## Discussion and conclusions

A correlation between sarcoidosis and carcinogenesis remains unproven. Since the original report by Brincker and Wilbek describing an increased prevalence of various malignancies in the context of sarcoidosis [2], investigators have sought to characterize a causal relationship based on the hypothesis that the altered immune response in sarcoidosis may predispose patients to cancer [10]. Malignancies linked to sarcoidosis include both hematological and solid malignancies, including breast cancer [2, 11]. Concomitant sarcoidosis and breast cancer has been described in case reports [12]. The frequency of breast cancer could be higher among patients with sarcoidosis than in the general population.

PET/CT has been recognized as a powerful imaging modality for assessing patients with primary breast cancer. This noninvasive, all-in-one imaging modality is useful in whole-body staging, restaging, and monitoring of treatment response in breast cancer patients [13–15]. However, false-positive FDG uptake or false-negative PET scans are frequent. Active granulomatous processes such as tuberculosis, fungal infections, and sarcoidosis have been reported to cause accumulation of FDG and can cause false-positive results. Acute or chronic infection, or inflammation must therefore always be considered, especially in patients with a diagnosis of cancer [16]. High FDG uptake in activated inflammatory cells is due to markedly increased glycolysis and the hexose monophosphate shunt that is stimulated by phagocytosis, resulting in increases of 20–30 times baseline values [16]. In cases of simultaneous sarcoidosis and malignancy, FDG-PET/CT may be the only additional diagnostic tool used to assess the extent of disease spread. In such cases, if malignant and granulomatous disorders are not differentiated, the results could create diagnostic difficulties and misunderstandings [17, 18]. Only histological verification should be relied on for accurate diagnosis when lesions showing FDG uptake cannot be confirmed as benign.

The diagnosis of sarcoidosis is not standardized, but is based on three major criteria: a compatible clinical presentation, the finding of nonnecrotizing granulomatous inflammation in one or more tissue samples, and the exclusion of alternative causes of granulomatous disease [19]. In this case, the tissue biopsy from one lymph node was performed; several lymph nodes tissue biopsy rather than cytology should be examined for making a definitive diagnosis. Besides systemic sarcoidosis, noncaseating epithelioid cell granulomas on histopathological examination have also been observed, not only in various kinds of parenchyma but also in lymph nodes associated with other granulomatous diseases. These lesions are caused by infections, environmental exposure to chemical substances, autoimmune disorders, and malignant diseases and have been termed “sarcoid reactions” or “sarcoid-like reactions” [20–22]. The incidence of sarcoid reaction was reported as 2.2% in patients with breast cancer by Giunti *et al.* [23]. The mechanism underlying tumor-associated sarcoid reaction in regional nodes has yet to be elucidated. Some authors have suggested the following relationship between malignant tumors and sarcoid reactions: (1) a localized defense reaction to tumor cells themselves, (2) a simple tissue reaction to tumor embolism into the lymphatic system or capillaries, or (3) an immunological reaction to substances released from tumors transported along the lymphatic system [24, 25]. Diagnose of sarcoidosis requires confirmation of epithelioid granulomas in tissues that are not close to malignant tumors and consideration of other systemic symptoms and laboratory findings [26].

This report emphasizes the importance of differential diagnosis of lymph node involvements in cancer patients. Furthermore, clinical information associated with systemic disease should be obtained. In this case, a history of uveitis 5 years earlier was considered a symptom of sarcoidosis. Although multiple lymph node metastases were suspected from FDG-PET/CT, EBUS-TBNA revealed the lymphadenopathy of hilar lymph nodes represented noncaseous epithelial granulomas due to sarcoidosis and the breast cancer was downgraded from stage IV to stage IIA. Despite mediastinoscopy having been considered the best option for some time, to this day, EBUS-TBNA represents the first choice for invasive mediastinal staging, according to international recommendations from the American College of Chest Physicians (ACCP) [27–29], European Society of Thoracic Surgeons (ESTS) [30], National Comprehensive Cancer Network (NCCN) [31], and European Society for Medical Oncology (ESMO) [32]. EBUS-TBNA provides a valid, minimally invasive alternative to mediastinoscopy and anterior mediastinotomy with equivalent diagnostic accuracy, more excellent safety, and lower total medical costs [33]. EBUS-TBNA has also been reported to be a useful and safe procedure for diagnosing sarcoidosis with lymphadenopathy of the mediastinum and hilum [34–37].

In conclusion, we report a case of breast cancer in which concomitant sarcoidosis was diagnosed based on EBUS-TBNA, a history of uveitis, and elevated sIL-2R levels despite suspicion of multiple lymph node metastases from FDG-PET/CT. Many cases of breast cancer and sarcoidosis have been reported and require close examination. In the present case, the patient completed preoperative chemotherapy and surgery according to the precise diagnosis without any progression of sarcoidosis.

## Data Availability

The data presented in this study are available in this article.

## Abbreviations

EBUS-TBNA: Endobronchial ultrasound-guided transbronchial needle aspiration
MRI: Magnetic resonance imaging
PET/CT: Positron emission tomography/computed tomography
FDG: Fluorodeoxyglucose
SUV: Standard uptake value
